# Mental Health during the COVID-19 Crisis in Africa: A Systematic Review and Meta-Analysis

**DOI:** 10.3390/ijerph182010604

**Published:** 2021-10-10

**Authors:** Jiyao Chen, Nusrat Farah, Rebecca Kechen Dong, Richard Z. Chen, Wen Xu, Jin Yin, Bryan Z. Chen, Andrew Yilong Delios, Saylor Miller, Xue Wan, Wenping Ye, Stephen X. Zhang

**Affiliations:** 1 College of Business, Oregon State University, Corvallis, OR 97330, USA; jiyao.chen@oregonstate.edu (J.C.); millesay@oregonstate.edu (S.M.); 2 College of Business and Analytics, Southern Illinois University Carbondale, Carbondale, IL 62901, USA; nusrat.farah@siu.edu; 3 Business School, University of South Australia, Adelaide, SA 5001, Australia; Rebecca.dong@unisa.edu.au; 4 Crescent Valley High School, Corvallis, OR 97330, USA; richardziychen@gmail.com (R.Z.C.); chenzbryan@gmail.com (B.Z.C.); 5 International Business and Management Department, Nottingham University Business School China, University of Nottingham Ningbo China, Ningbo 315100, China; wen.xu@nottingham.edu.cn; 6 School of Humanities, Southeast University, Nanjing 211189, China; allen-yin@hotmail.com; 7 Department of Psychology, University of Adelaide, Adelaide, SA 5001, Australia; delios9580@gmail.com; 8 School of Economics and Management, Tongji University, Shanghai 200092, China; wanxue@tongji.edu.cn; 9 Department of Business Administration, School of Management, Jinan University, Guangzhou 510632, China; yzq721@yeah.net; 10 Faculty of Professions, Entrepreneurship, Commercialization and Innovation Center, University of Adelaide, Adelaide, SA 5005, Australia

**Keywords:** mental health, prevalence, pandemic, general population, healthcare workers, anxiety, depression, Insomnia

## Abstract

We aim to provide a systematic review and meta-analysis of the prevalence rates of mental health symptoms among major African populations during the COVID-19 pandemic. We include articles from PubMed, Embase, Web of Science, PsycINFO, and medRxiv between 1 February 2020 and 6 February 2021, and pooled data using random-effects meta-analyses. We identify 28 studies and 32 independent samples from 12 African countries with a total of 15,071 participants. The pooled prevalence of anxiety was 37% in 27 studies, of depression was 45% in 24 studies, and of insomnia was 28% in 9 studies. The pooled prevalence rates of anxiety, depression, and insomnia in North Africa (44%, 55%, and 31%, respectively) are higher than those in Sub-Saharan Africa (31%, 30%, and 24%, respectively). We find (a) a scarcity of studies in several African countries with a high number of COVID-19 cases; (b) high heterogeneity among the studies; (c) the extent and pattern of prevalence of mental health symptoms in Africa is high and differs from elsewhere—more African adults suffer from depression rather than anxiety and insomnia during COVID 19 compared to adult populations in other countries/regions. Hence, our findings carry crucial implications and impact future research to enable evidence-based medicine in Africa.

## 1. Introduction

Africa, a huge continent that is home to 1.37 billion people, is particularly vulnerable to the highly contagious COVID-19 disease due to its unique and severe limitations [1,2]. Africa has limited medical facilities and resources [3], such as the lack of advanced healthcare facilities [4] and intensive care units, understaffed and overcrowded hospitals, crippling healthcare coordination and transportation, limited access to sanitary items and clean water [5], inefficient primary healthcare infrastructure [6], limited vaccinology training programs [7], and limited vaccine penetration in its fight against the COVID-19 pandemic. The COVID-19 pandemic threatens to further constrain the limited medical services and African peoples’ access to mental healthcare. A number of studies have started researching the mental health conditions of Africans under the COVID-19 pandemic. For example, a study in Ethiopia found that people using public transportation had developed and experienced general anxiety disorder [8], and research showed a significant number of healthcare workers in Libya had low levels of knowledge, awareness, and preparedness for COVID-19 [9]. Such emerging evidence suggests the importance of studying mental health symptoms in Africa during the COVID-19 pandemic [10]. 

However, the emerging evidence on mental health symptoms during the COVID-19 pandemic in Africa has reported a heterogeneous set of findings. For example, one study found that 56.3% of healthcare workers in Libya had depressive symptoms [11]; another study found the prevalence of depression in Ethiopia to be 21.2% [12]. To understand the prevalence of mental health symptoms across the studies, several meta-analyses have been conducted in other regions during the COVID-19 pandemic [13,14,15,16,17,18,19], but not in Africa, one of the least developed and least understood continents. 

This study aims to address this gap by providing a systematic review to estimate the pooled prevalence of anxiety, depression, and insomnia in Africa during the COVID-19 pandemic. This systematic review and meta-analysis of studies on Africa helps to guide practitioners and mental health researchers during this continued global pandemic in several ways. First, we benchmark and compare the pooled prevalence of mental health symptoms in Africa vs. those reported by meta-analyses in other regions. Second, we assess the pooled prevalence rates of anxiety, depression, and insomnia in Sub-Saharan Africa vs. North Africa to reveal heterogeneities within Africa. Third, we pool and compare the prevalence among the key populations studied so far, including frontline healthcare workers (HCWs), general HCWs, general population, and medical students. The findings reveal unique patterns in Africa to guide psychiatric organizations such as the World Psychiatric Association (WPA) in their effort to improve/understand/address mental health services in Africa. 

## 2. Methodology and Materials

### 2.1. Protocol Registration

To gain an understanding of the prevalence of mental health symptoms during COVID-19 in Africa, we conducted a systematic review and meta-analysis in accordance with the Preferred Reporting Items for Systematic Reviews and Meta-Analyses (PRISMA) statement 2020 and registered in the International Prospective Register of Systematic Reviews (PROSPERO: RD42020224458). Please see Appendix A for the PRISMA Checklist.

### 2.2. Data Sources and Search Strategy 

This paper is part of a large project to examine the prevalence of mental health symptoms during the COVID-19 pandemic in key geographical areas. We performed a comprehensive literature search in the databases of *PubMed*, *Embase*, PsycINFO, *Web of Science*, and *medRxiv* from 1 February 2020 to 6 February 2021. We used the search query reported in Appendix Table A1 and entered keywords in each database using Boolean operators within the titles, abstracts, keywords, and subject headings (for example, MeSH terms). 

### 2.3. Eligibility Criteria 

In this systematic review, we included original empirical studies that examined the impact of the COVID-19 pandemic on adults’ mental health in Africa if they met the following criteria:

- Outcomes: reported the prevalence of depression, anxiety, or insomnia during COVID-19 pandemic.
- Measurements/Instruments: using any validated measurement tools or scales.
- Population: included adult participants including frontline HCWs, general HCWs, general adult population, or adult medical students from any African countries.
- Methodological design: cross-sectional or cohort.
- Language: published in English.

We excluded studies based on the following criteria:

- Population: children, adolescents, or specific niche adult populations, such as patients, adults under quarantine, pregnant/postpartum women.
- Methodological design: non-primary studies, such as reviews or meta-analyses, qualitative or case studies without a validated instrument, interventional studies, interviews, or news reports.
- Measurements/Instruments: non-validated instruments measuring mental health outcomes (i.e., self-made questionnaire) or instruments missing a validated cutoff score to calculate the prevalence rate.

A researcher (W.X.) contacted the authors of papers that missed important information. For example: (1) if the surveyed population in the paper included both targeted and excluded populations in a way the prevalence rate of the relevant population cannot be identified; (2) if the prevalence rates were not reported; (3) if overall prevalence rates were reported without specifying cutoff points to determine whether it is mild above or moderate above; or (4) if the paper lacks any important information, such as response rate, time frame of data collection, sample size, or gender proportions.

### 2.4. Screening and Data Extraction

One researcher (J.C.) exported the included articles from the databases into an EndNote library where we identified duplicates and then imported the articles to Rayyan for screening. Two researchers (B.Z.C. and A.D.) independently screened the imported articles based on their titles and abstracts, and a third researcher (R.K.D.) resolved any conflicts. Six coders, in pairs (W.X. and J.Y., B.Z.C. and A.D., R.Z.C. and S.M.), assessed the eligibility of each paper to be included in the review. They read the full texts to extract the relevant data into a coding book based on a coding protocol; both were developed in a previous study [20]. The coding book records all the coded information, such as authors’ names, year of the publication, title, publication status, sample locations, date of data collection, sample size, response rate, population, age (mean, SD, min, and max), gender proportion, instruments, cutoff scores used, and the prevalence/mean/SD of the mental health outcomes. Each paper was double-coded and crosschecked independently by a pair of coders. The pair discussed any discrepancies. In cases where the pair of coders failed to resolve the discrepancies, a third coder (R.K.D.) checked the paper independently to determine its coding. Another coder (R.Z.C.) double-checked important data including the population, sample size, mental health outcomes, outcome levels, instruments, and prevalence, and further checked papers with unusual prevalence, cutoff scores, and numbers for sensitivity analysis.

### 2.5. Assessment of Bias Risk

We followed prior meta-analyses [21,22] by using the Mixed Methods Appraisal Tool (MMAT) [23,24,25,26] with seven questions to gauge the quality of the studies included in the meta-analysis. We selected MMAT as it is adjudged to be the most comprehensive and best approach available for evaluating multi-method studies [21,27]. A pair of coders individually evaluated the risk of non-response bias and assessed the quality based on the representativeness of sample, appropriateness of measurements/instruments using the MMAT. Each article was assigned a final quality score ranging from 0 to 7. Articles with a quality score higher than 6 were considered low bias risk, articles with a score between 5 and 6 were classified as medium bias risk, and articles with a score below 5 were classified as high bias risk. Any discrepancies regarding the quality scores were resolved by a discussion between the pair of coders or the lead researcher.

### 2.6. Statistical Analysis

The overall prevalence and 95% confidence intervals of mental health symptoms were pooled using Stata 16.1. Following prior literature on the prevalence of mental health symptoms, the random-effects model was used to extract the pooled estimates [28]. The random-effects model assumes that the included studies in the meta-analysis are random samples from a large population to generalize findings beyond the chosen studies [29]. We also performed subgroup analyses by the key potential sources of heterogeneity of outcomes (three types of mental health symptoms), the severity of outcome (above mild/above moderate/above severe), four major population groups (frontline HCWs, general HCWs, general population, students), and two regions in Africa (Sub-Saharan vs. Northern Africa).

## 3. Results

### 3.1. Study Screening

**Figure 1:**
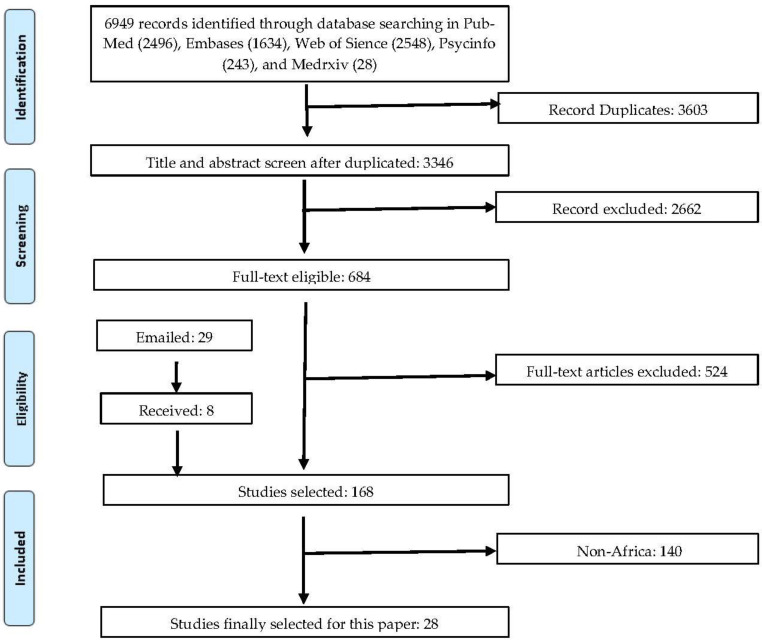
A PRISMA flow diagram.

As illustrated in Figure 1, our search in the selected databases and other sources yielded 6949 papers. Removing the duplicates, we examined the titles and abstracts of the remaining 3346 papers and excluded 2662 of them that did not meet the inclusion criteria in the screening process. By examining the full text of the remaining 684 pa-pers, we identified 150 papers relevant to the meta-analyses. However, 95 papers missed certain information. We sent out two rounds of emails to their authors to re-quest the missing information, and 75 of them responded, adding 8 sets of prevalence data. The final sample contains 168 studies with the information required for the me-ta-analysis. Among these 168 studies, 28 articles involve participants from Africa.

### 3.2. Study Characteristics

In this meta-analysis, we included 28 [8,11,12,30,31,32,33,34,35,36,37,38,39,40,41,42,43,44,45,46,47,48,49,50,51,52,53,54] articles that contain 32 samples and 137 individual prevalence rates from a total of 15,071 individual participants. Appendix Table A3 lists the 28 articles included in this meta-analysis. Table 1 summarizes the key characteristics of the included papers.

Among the 32 independent samples, 9.4% studied frontline HCWs, 37.5% studied general HCWs, 46.9% studied the general population, and 6.3% studied medical students. Sample prevalence was 45.3% for anxiety, 39.4% for depression, and 15.3% for insomnia. Respectively, 40.2%, 32.9%, 24.1%, and 2.8% of prevalence reported at the mild above, moderate above, severe above, and overall level by the severity of the symptoms. Cross-sectional surveys comprised 96.7% of the studies while 3.3% used cohort-based studies. All the articles were published in journals. The median number of individuals per sample was 327 (range: 48 to 2430) with a median female proportion of 56.6% (range: 0% to 100%) and a median response rate of 49.0% (range: 18.2% to 94.2%).

### 3.3. Pooled Prevalence Rates of Mental Health Symptoms

The prevalence rates of the 32 samples were pooled by the subgroups (Table 2). 

**Figure 2:**
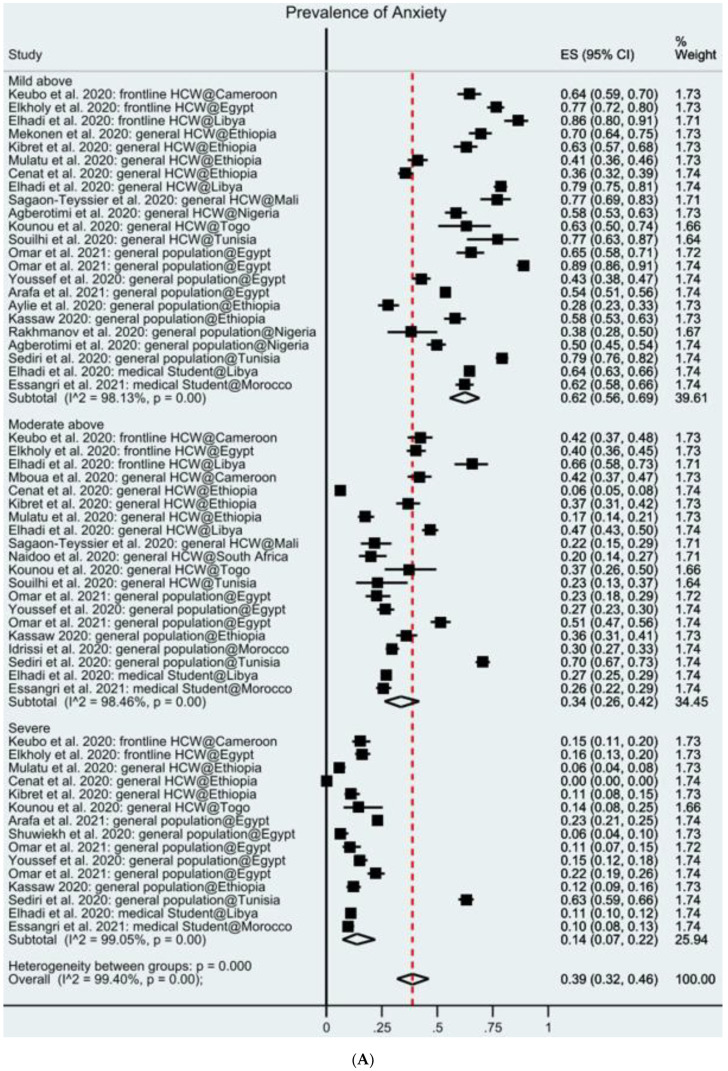

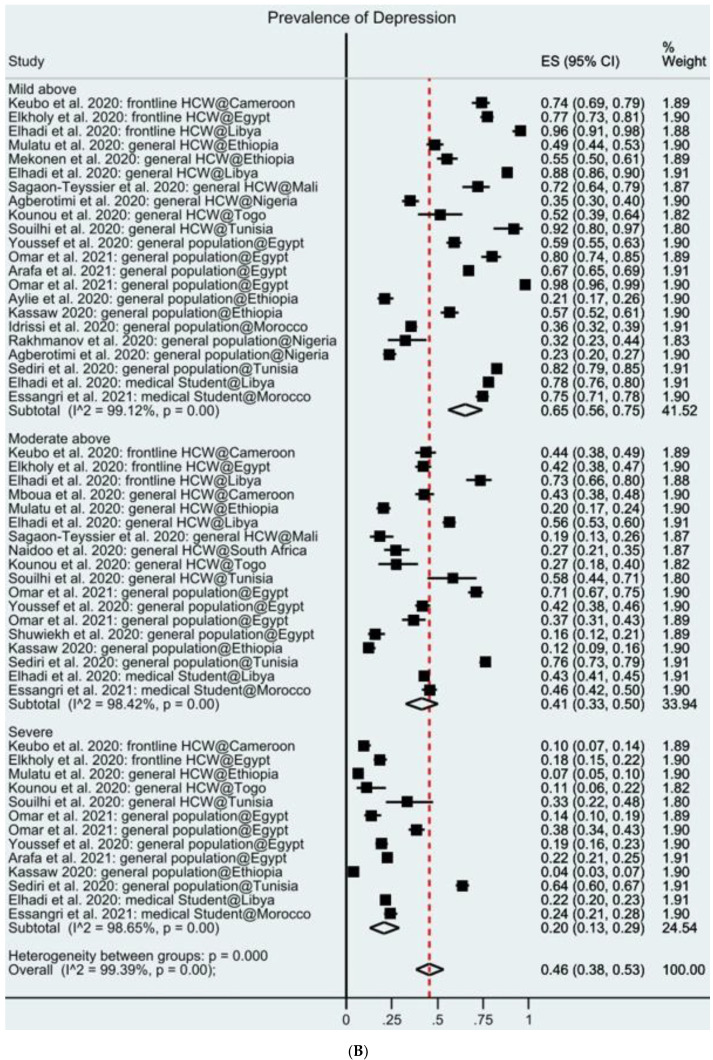

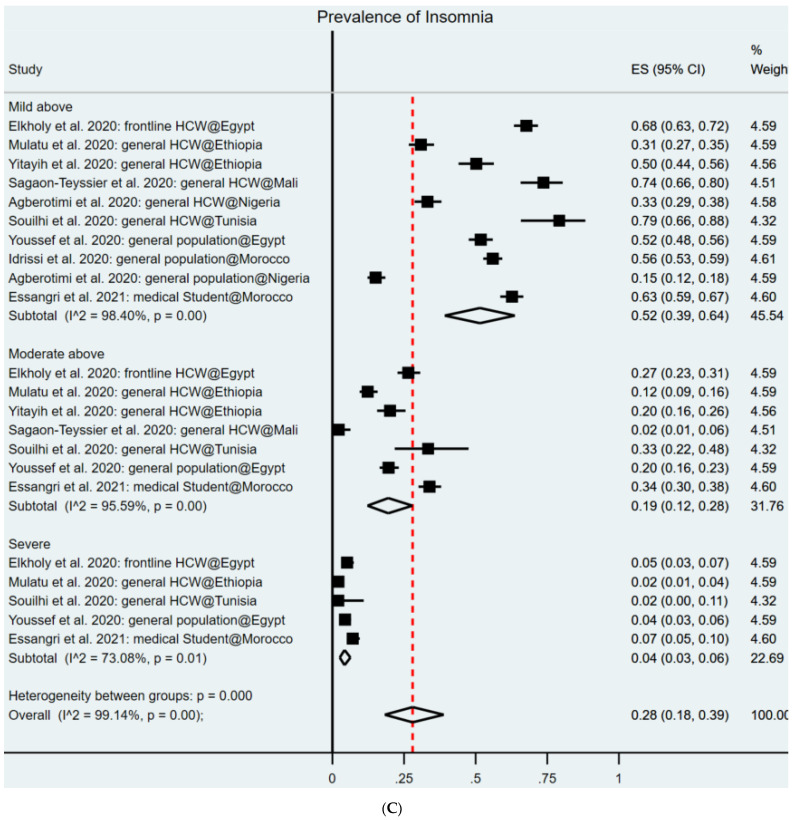
(**A**) Forest plot of the prevalence of anxiety. (**B**) Forest plot of the prevalence of depression. (**C**) Forest plot of the prevalence of insomnia. Figure legend: the square markers indicate the prevalence of insomnia at the different levels for different populations. The size of the marker correlates to the inverse variance of the effect estimates and indicates the weight of the study. The diamond data markers indicate the pooled prevalence.

The overall prevalence of mental health symptoms is 39% in Africa; 31 samples from 27 studies reported the prevalence of anxiety symptoms among 14,847 participants. Several anxiety instruments were used, with the Generalized Anxiety Disorder 7-items scale (GAD-7) used the most (51.8%), followed by the Depression, Anxiety and Stress Scale—21 Items (DASS-21) (22.2%), Hospital Anxiety and Depression Scale (HADS) (14.8%), Hamilton Anxiety Rating (HARS) (3.7%), Hopkins Symptoms Checklist (HSCL) (3.7%), Self-Reporting Questionnaire (SRQ) (3.7%). In the random-effects model, the pooled prevalence of anxiety was 37% (95% CI: 31–44%) in the 23 studies (Figure 2A). This finding suggests that, on average, 37% of the adults in Africa during COVID-19 had anxiety symptoms. The variance of true effects (tau2) across studies is 0.30. The prediction internal is 1% to 91% based on a normal distribution; hence, we expect that the prevalence of anxiety symptoms in any comparable studies will fall in this range.

Samples of our total 24 articles that we reported in this meta-analysis were on depression, for a total of 12,688 respondents. Several depression instruments were used, including Patient Health Questionnaire (PHQ)-9 (45.8%), DASS-21 (25.0%), HADS (16.6%), Beck Depression Inventory (BDI) (4.2%), CES-D (4.2%), and SRQ (4.2%). In the random-effects model, the pooled prevalence of depression was 45% (95% CI: 38–53%) among the 24 studies (Figure 2B). This finding suggests that, on average, 45% of the adults in Africa during COVID-19 had depression symptoms. The variance of true effects (tau2) across studies is 0.31 with a prediction internal from 1% to 99%.

Ten samples of the nine articles that we reported in this meta-analysis studied insomnia, for a total of 4144 respondents. The Insomnia Severity Index (ISI) (88.9%) and Athens Insomnia Scale (AIS) (11.1%) were used to measure insomnia. In the random-effects model, the pooled prevalence of insomnia is 28% (95% CI: 18–39%). This finding suggests that on average 28% of the adults in Africa during COVID-19 had insomnia symptoms. The variance of true effects (tau2) across studies is 0.30 with a prediction internal from 0.1% to 70%.

The overall prevalence rates of mental health symptoms that surpassed the cutoff values of mild, moderate, and severe symptoms were 62%, 34%, and 14%, respectively. The overall prevalence of mental health symptoms in frontline HCWs, general HCWs, general population, and medical students in African countries are 49%, 36%, 38%, and 38%, respectively. The range of I^2^ is 98.4–99.1%, which is similar to those in the published meta-analyses on the same topic [13,14,15,16,17,19]. By geographical regions, the prevalence of mental health symptoms is higher in North Africa (46%) than in Sub-Saharan Africa (30%). By time frame, the prevalence of mental health symptoms is higher in the first six months of the COVID-10 pandemic (November 2019 to April 2020, 42%) than in the subsequent period (26%).

### 3.4. Study Quality

Overall, 6 studies (21.4%) are of higher quality, 22 studies (78.8%) have a medium quality, and none of the studies have low quality. Subgroup analysis suggests the studies with high quality (51%) reported a significantly higher prevalence of mental health symptoms in Africa than studies with medium quality (37%) (Table 2).

### 3.5. Sensitivity Analysis

**Figure 3:**
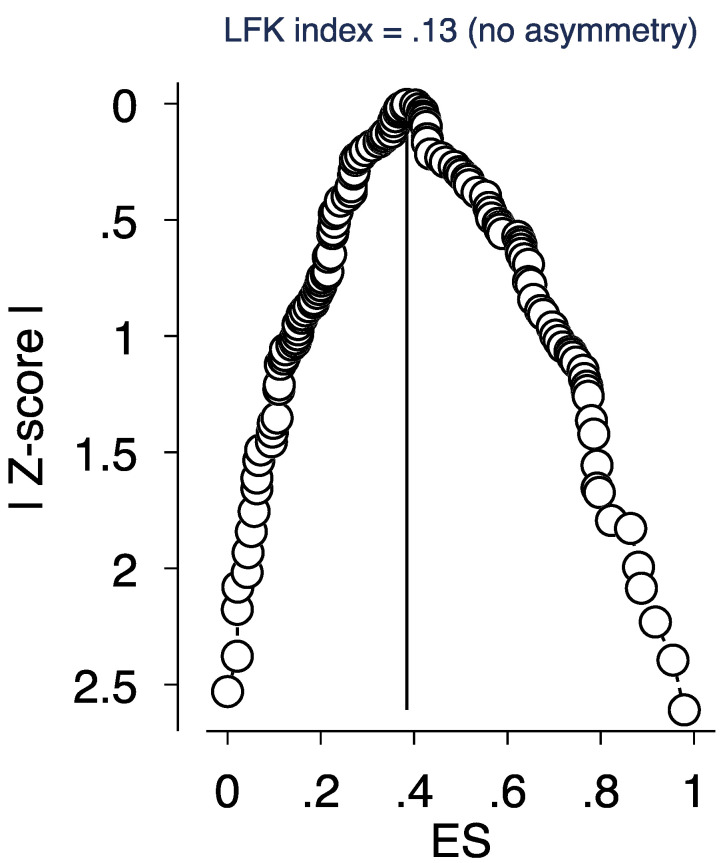
DOI plot and the Luis Furuya–Kanamori (LFK) index. Figure legend: depiction of publication bias in the baseline meta-analysis of proportion studies based on a DOI plot and the Luis Furuya–Kanamori (LFK) index—a score that is within ±1 indicates no asymmetry.

Conventional funnel plots have been found to be inaccurate for proportion study meta-analyses [55]. Due to higher sensitivity and power, a better approach for publication bias graphical representation is a DOI plot in combination with the Luis Furuya–Kanamori (LFK) index rather than using a funnel plot and Egger’s regression [44,45]. Asymmetry is assessed quantitatively through the LFK index. Scores are ±1, between ±1 and ±2, or ±2, indicating no asymmetry, minor asymmetry, and major asymmetry, respectively. No asymmetry is depicted in Figure 3, indicating a DOI plot and LFK index of 0.13. Therefore, the presence of publication bias is unlikely. Further sensitivity analysis was conducted on publication status and sample size; no significant influence was found. 

## 4. Discussion

### 4.1. Comparisons of Results with Prior Meta-Analyses

Our meta-analytical findings revealed several crucial pieces of evidence on the prevalence of mental health symptoms during the COVID-19 crisis in Africa. Given this study is the first such meta-analysis, it would be useful to compare our findings from Africa with some published meta-analyses in other regions. The meta-analytical evidence shows more African adults suffered from depression (45%) and anxiety (37%) than insomnia (28%)—a pattern different from those in other countries. For example, more Chinese adults suffered from insomnia (19%) than anxiety (11%) and depression (13%) [20], and more Spanish adults suffered from insomnia (52%) than anxiety (20%) and depression (23%) [56]. 

The extant meta-analyses on COVID-19 mental health covered very limited regions, mostly in China and several other developed countries. Moreover, the extant meta-analytical evidence covered the literature published before May 2020, which was at the early onset of the COVID-19 pandemic. We include articles published by February 2021 to cover more recent findings and enable better accumulative evidence on the prevalence of mental health symptoms in Africa. 

Our finding on the pooled prevalence rates of anxiety in Africa mostly exceeded the reported prevalence rates by meta-analyses in other geographical areas. The pooled prevalence rate for anxiety in the African population is 37%, which is significantly higher than those in China reported in Bareeqa et al. (2020) (22%; *p*-value < 0.0001), Pappa et al. (2020) (23%; *p*-value < 0.0001), Krishnamoorthy et al., 2020 (26%; *p*-value < 0.0001), and Ren et al., 2020 (25%; *p*-value < 0.0001) [13,14,15,16], and those in Spain (20%; *p*-value < 0.0001) [56]. However, notably, the pooled prevalence rate for anxiety in South Asian countries is significantly higher than that in Africa (41.3%; *p*-value < 0.0001) [57]. In addition, the pooled prevalence of anxiety in Africa (37%) is higher than those in individual cross-country individual studies, such as a study of 10 countries (China, India, Japan, Iran, Iraq, Italy, Nepal, Nigeria, Spain, and the UK) (32%; *p*-value < 0.0001) [19] and a study in 17 countries in the regions of Asia (China, India, Japan, Pakistan, Singapore, Vietnam), Middle East (Iran, Israel), Europe (Denmark, Greece, Italy, Spain, Turkey), and Latin America (Argentina, Brazil, Chile, and Mexico) (33%; *p*-value < 0.0001) [58]. Moreover, we find that the pooled prevalence rate of anxiety among frontline HCWs in Africa (51%) is significantly higher than Bareeqa et al. (2020) (24%; *p*-value < 0.0001), Krishnamoorthy et al., 2020 (26%; *p*-value < 0.0001), and Ren et al., 2020 (27%; *p*-value < 0.0001) [13,15,16]. Similarly, we find that the pooled prevalence rate of anxiety among the general population (37%) in Africa is significantly higher than Ren et al., 2020 (24%; *p*-value < 0.0001) [15]. 

Our finding on the pooled prevalence rates of depression in Africa exceeded the reported prevalence rates by meta-analyses in most of the other geographical areas. The pooled prevalence rate for depression in the African population (45%) is significantly higher than those in China reported by Bareeqa et al. (2020) (27%; *p*-value < 0.0001), Pappa et al. (2020) (23%; *p*-value < 0.0001), Krishnamoorthy et al., 2020 (26%; *p*-value < 0.0001), and Ren et al., 2020 (28%; *p*-value < 0.0001) [13,14,15,16]. The pooled prevalence rate for depression in the African population (45%) is higher than those in Spain (23%; *p*-value < 0.0001) [56] and in South Asian countries reported by Hossain et al. (2020) (34%; *p*-value < 0.0001) [57]. The pooled prevalence for depression in Africa (45%) is also higher than the pooled prevalence in a study including over 17 countries reported by Luo et al. (28%; *p*-value < 0.0001) [58] and another study of 10 countries reported by Salari et al. (34%; *p*-value < 0.0001) [19]. However, the prevalence rate for depression in Africa is lower than Italy—the country with the highest prevalence for depression (67%) [58]. Furthermore, we find that the pooled prevalence rate of depression among frontline HCWs (55%) is significantly higher than Bareeqa et al. (2020) (32%; *p*-value < 0.0001), Krishnamoorthy et al., 2020 (25%; *p*-value < 0.0001), and Ren et al., 2020 (25%; *p*-value < 0.0001). Moreover, the pooled prevalence rate of depression among the general population (42%) in Africa is significantly higher than Krishnamoorthy et al., 2020 (24%; *p*-value < 0.0001). 

The pooled prevalence rate for insomnia in the African population (28%) is similar to those in China [20], but significantly lower than those in Spain (52%; *p*-value < 0.0001) [56], Germany (39%; *p*-value < 0.0001), Italy (55%; *p*-value < 0.0001), and France (51%; *p*-value < 0.0001), as well as the pooled prevalence in the 13 countries, including Australia, Bahrain, Canada, Germany, Greece, Iraq, India, Mexico, and the USA (36%; *p*-value < 0.0001), as reported by Jahrami et al. (2021) [59].

The comparisons above show that the pooled prevalence rates for the African population are significantly higher than in the populations in most other regions, suggesting that the mental health symptoms under COVID-19 may not be uniform across regions or countries, and it is worthwhile for individual studies to explore the mental health conditions around the globe and for meta-analytical studies to pool the prevalence across regions. 

The subgroup analysis results that the pooled prevalence rates of anxiety, depression, and insomnia in Sub-Saharan Africa (31%, 30%, and 24%, respectively) are significantly lower than those reported in North Africa (44%, 55%, and 31%, respectively) suggest a high heterogeneous prevalence of mental health symptoms within the mega-regions of Africa (see Appendix Table A2). The prevalence rate differences between Sub-Saharan Africa and North Africa may arise due to lack of awareness of the danger of COVID-19 due to insufficient COVID-19 testing or the lower death rates due to the younger population in Sub-Saharan Africa [60]. Our results suggest future research can further examine why such heterogeneity in the prevalence rates for the mental health symptoms occur between Sub-Saharan Africa and North Africa.

### 4.2. Comparisons of COVID vs. Pre-COVID Findings in Africa

To assess the prevalence of mental health symptoms during COVID-19, we benchmark the meta-analytical findings with the pre-COVID estimates of mental health symptoms in several African countries. For example, in Egypt, the prevalence of depressive symptoms among university students in 2004 was 71% (95% CI  =  67.1–72.8) with 28.2% (95% CI  =  25.4–36.9) suffering from moderate depression and 8.8% suffering from severe depression (95% CI  =  7.1–10.7) [61], and the overall prevalence of mental health disorders in a representative sample of adults from five regions in Egypt in 2003 was 16.93% [62]. The prevalence of mental distress among working adults in Addis Ababa, Ethiopia between December 2009 and January 2010 was 17.7% [63], while the prevalence of common mental disorder symptoms among adults in rural Ethiopia was 27.9% (95% CI  =  25.6–30.2), with mild, moderate, and severe thresholds at 13.8%, 9.0%, and 5.1%, respectively [64]. The prevalence of common mental disorders among adults in Kombolcha town in Ethiopia from March to April 2013 was 32.4% (95% CI = 30.3–34.5) [65]. In a representative sample of the Moroccan general population, 26.5% suffered from depressive disorder and 37% suffered from at least one anxiety disorder [66], while the prevalence rate of anxiety disorder was 25.5% [67]. In Nigeria, the prevalence of depression in a community sample from Oyo state was found to be 5.2% [68], while the prevalence rates of depressive and anxiety disorders from Ebonyi state were 70% and 85.3%, respectively [69]. Among Nigerian medical students from the University of Ibadan, the prevalence of anxiety and depression was 26.5% and 10.1%, respectively [70]; from Enugu State University of Science and Technology, the prevalence of anxiety disorders was 14.3% [71]. In a nationally representative sample of South African adults between 2002 and 2004, 4.9% suffered from major depressive disorder and 8.1% suffered from any anxiety disorder [72]. A recent population-based study on adults from six of eleven South African provinces documented that 47.3% and 17.7% of the surveyed adults suffered from minimal or mild depression and moderate to severe depression, respectively [73]. In adults from Rwanda over a three-month period in 2008, the overall prevalence of depression and PTSD was 22.7% and 26.1%, respectively [74]. From a nationally representative sample in Ghana in 2009–2010, the prevalence of psychological distress was 18.7% with 11.7% and 7.0% suffering from moderate and severe psychological distress, respectively [75]. In urban Ghana in 2010, the prevalence of depressive disorder and mental distress was 12.7% and 29.88%, respectively [76]. These higher pre-COVID baseline estimates of prevalence of mental health symptoms across African countries corroborate our meta-analytical results, which, in a consistent manner, find the prevalence to be on the high side among various African populations. We encourage future research to continue monitoring mental health symptoms in the African population to gather more evidence as COVID-19 continues.

### 4.3. Practical Implications of Our Findings for Psychiatrists/Healthcare Organizations 

Our meta-analysis put forward evidence of a high proportion of mental health symptoms among the general population and healthcare workers (HCWs) during COVID-19 in Africa. The fear, worry, and uncertainty surrounding such an unknown threat and containment strategies can put a burden on mental health. The higher prevalence rates of depression in the African population compared to those reported elsewhere could be due to the higher baseline prevalence rates in Africa. A meta-analysis of studies published between 1 January 2006 and 31 July 2011, showed that the pooled prevalence rate of depression symptoms and major depression in Sub-Saharan Africa was 31% and 18%, respectively [77]. Between 2000 and 2015, while Africa’s population grew by 49%, some mental health and substance use disorders increased by 52% [78]. Making issues worse, mental health symptoms received less attention in underdeveloped economies [79]; for instance, less than 10% of people suffering from depression in low-resource settings have access to mental health treatment [80] despite depression being one of the major causes of disability. The vast majority of Africa suffers from lack of affordable oxygen supply in hospitals [81], high prevalence of multimorbidity [82], low-income, high mortality rates, malnutrition, high incidence of infectious diseases, poor health facilities [83,84], specifically mental health resources, programs, or facilities, [79], which all can present unique situations under the COVID-19 pandemic, in terms of the mental health of the African population. 

Our finding that frontline HCWs suffered a much higher prevalence of mental health symptoms (49%) than other populations (general HCWs (36%), general population (38%), medical students (38%)) suggests that healthcare organizations need to pay special attention to providing frontline HCWs with proper mental and healthcare support during the COVID-19 crisis. Our finding of higher prevalence rates of depression (45%) than anxiety (37%) and insomnia (28%) in Africa—a pattern different from elsewhere—suggests not only the extent, but also the patterns of mental health issues could be unique in Africa, or at least vary across geographical regions. As such, psychiatric organizations, such as the World Psychiatric Association (WPA), need to consider the mental health in African countries differently to customize mental health services. 

### 4.4. Limitations and Future Work

Our meta-analysis is not free of limitations. First, the validity of our findings depends on the quality and reporting of the original studies included in the meta-analysis. The individual mental health studies employed a variety of instruments, cutoff scores, levels of cutoff scores to classify the severity of mental illness, and various reporting standards. For example, many studies report the overall prevalence rates without specifying which/how cutoff scores are utilized. While we tried to reduce the additional noise and variance in the meta-analysis by paying extra attention to the severity, cutoff points, and the manners in which individual articles utilized and reported cutoff points, the profusion of different practices can lead to some biases and noise. Second, this systematic review identified empirical studies from only 12 out of the 48 Africa countries. No studies have appeared on the mental health of people in three quarters of African countries, even though those African countries are not immune to the COVID-19 pandemic. Hence, we call for research to investigate the mental health in Africa, the least studied continent with most of the countries not studied at all. A possible reason is that we included articles published in English, which may result in some biases. Third, 96.7% of the studies included in this meta-analysis conducted cross-sectional surveys, whereas only 3.3% of the articles used cohort studies. We believe scholars need to focus more on cohort-based studies to examine mental health symptoms during COVID-19 over time to further our understanding regarding how a highly contagious disease can affect psychological well-being. Finally, we only focus on studies that collected data in African countries, and we call for future meta-analyses in other countries or regions, as the COVID-19 pandemic is unfolding around the globe. 

## 5. Conclusions

This paper presents, to the best of our knowledge, the first systematic review and meta-analysis of the prevalence of mental health symptoms during the COVID-19 crisis in Africa. The findings provide evidence that the prevalence of anxiety and depression is much higher in the African population compared to those reported elsewhere. Given our findings and the unique challenges Africa faces under COVID-19, we call for more studies on mental health in Africa.

### Disclosure Statement

All authors have completed the Unified Competing Interest form and declare: no support from any organization for the submitted work that may create conflict of interest; no financial relationships with any organizations that might have an interest in the submitted work in the previous three years, no other relationships or activities that could appear to have influenced the submitted work. 

## Figures and Tables

**Table 1:** Characteristics of the studies on mental health in Africa during COVID-19 pandemic.

Characteristics	Total Number of Studies/Samples *	Percent (%)	Number of Participants	Percent (%)	Level of Analysis
*Overall*	28/32	100	15,071	100	
*Population*	32	100			Sample
Frontline HCWs	3	9.4	919	6.1	
General HCWs	12	37.5	3928	26.1	
General population	15	46.8	7245	48.1	
Medical students	2	6.3	2979	19.8	
*Outcome #*	137				Prevalence
Anxiety	62	45.3	14,847	98.5	
Depression	54	39.4	12,688	84.2	
Insomnia	21	15.3	4144	27.5	
*Severity #*	137				Prevalence
Above mild	55	40.2	6174	41.0	
Above moderate	45	32.9	3511	23.3	
Above severe	33	24.1	4133	27.4	
Overall	4	2.8	1253	8.3	
*Sampling Country*	32				Sample
Cameroon	2	6.3	624	4.1	
Egypt	6	18.	3593	23.8	
Ethiopia	7	21.8	2778	18.4	
Libya	3	9.3	3329	22.1	
Mali	1	3.1	135	0.9	
Morocco	2	6.3	1395	9.3	
Nigeria	3	9.3	953	6.3	
RDC	1	3.1	626	4.2	
Rwanda	1	3.1	174	1.2	
South Africa	2	6.3	361	2.4	
Togo	2	6.3	304	2.0	
Tunisia	2	6.3	799	5.3	
*Quality*					Article
High	3	21.4	2621	17.4	
Medium	22	78.6	12,450	82.6	
*Design*					Article
Cohort	1	3.3	211	1.4	
Cross-sectional	27	96.7	14,860	98.6	
*Data collection time*					Article
The first six months	22	80.2			
After the first six months	6	19.8	12,450	82.6	
	Mean (median)	Range	2621	17.4	
*Number of participants*	471 (327)	48–2430	-	-	Sample
*Female proportion*	53.1% (56.6%)	0–100%	-	-	Sample
*Response rate*	54.9% (49.0%)	18.2–94.2%	-	-	Sample

Notes: * Some studies include multiple independent samples. For example, Cénat et al., 2020 examined the prevalence of the general population in Togo, RDC, and Rwanda [43]. # One independent sample in an article may report anxiety, depression, and insomnia at the levels of mild above, moderate above, and severe. Hence, the total number of prevalence is larger than the total number of independent samples.

**Table 2:** The pooled prevalence rates of mental health symptoms by subgroups of population, outcome, and severity.

First-Level Subgroup	Second-Level Subgroup	Prevalence (%)	95% CI (%)	*p* Value *
Aggregated		39	34–44	<0.001
Outcome	Anxiety	37	31–44	<0.001
	Depression	45	38–53	<0.001
	Insomnia	28	18–40	<0.001
Population	Frontline HCWs	49	35–63	<0.001
	General HCWs	36	28–45	<0.001
	General population	38	31–45	<0.001
	Medical students	38	25–52	<0.001
Severity	Above mild	62	56–67	<0.001
	Above moderate	34	29–40	<0.001
	Above severe	14	10–20	<0.001
	Overall	17	09–27	<0.001
Region	Sub-Saharan Africa	30	24–36	<0.001
	North Africa	46	40 -53	<0.001
Quality	Studies with medium quality	51	40–63	<0.001
	Studies with high quality	37	32–42	<0.001
Data collection time	The first six months	42	36–47	<0.001
	After the first six months	26	17–35	<0.001

Note: CI = confidence interval. * The *p* value here refers to the significant test of mean prevalence.

## Data Availability

All data are secondary and available on request.

## Appendix A. PRISMA Checklist

**Table A1:** Search strategy used in this systematic review and meta-analysis.

Search Query	Search Topic	Search Keywords (Titles, Abstracts, and Subject Headings) with Boolean Operators
1	Exposure/Context	“Coronavirus” OR “COVID-19” OR “SARS-CoV-2” OR “2019-nCoV”
2	Outcome of interest	“Depression” OR “Depressive symptoms” OR “Depressive disorder*” OR “Anxiety” OR “Social Anxiety” or “Social Phobia” OR “Anxiety disorder*” OR “Insomnia” OR “Sleep disorder” OR “Depressive disorder*”
3	Epidemiological phenomenon	“Prevalence” OR “Incidence” OR “rate*” OR “ratio*” OR “Epidemiology*” OR “risk factor*” OR “relative risk” OR “disease burden”
4	Language	English
5	Time	February 2020–6 February 2021
	Intersection of four topics	1 AND 2 AND 3 AND 4 AND 5

**Table A2:** Subgroup analyses of the prevalence of anxiety, depression, and insomnia.

Groups	Subgroups	Anxiety	Depression	Insomnia
Number of studies		27	24	9
Number of samples		31	26	10
Number of prevalence		62	54	21
Number of participants		14,847	12,688	4144
Aggregated		37%, 95% CI: 31–44%	45%, 95% CI: 38–53%	28%, 95% CI: 18–40%
Population	Frontline HCWs	51%, 95% CI: 31–70%	55%, 95% CI: 32–76%	30%, 95% CI: 2–71%
	General HCWs	35%, 95% CI: 23–48%	43%, 95% CI: 39–58%	28%, 95% CI: 13–45%
	General population	37%, 95% CI: 28–46%	42%, 95% CI: 31–55%	27%, 95% CI: 9–50%
	Medical student	31%, 95% CI: 13–54%	48%, 95% CI: 27–69%	32%, 95% CI: 39–64%
Severity	Above mild	62%, 95% CI: 56–69%	65%, 95% CI: 56–75%	52%, 95% CI: 39–64%
	Above moderate	33%, 95% CI: 26–41%	41%, 95% CI: 33–50%	19%, 95% CI: 11–29%
	Severe	14%, 95% CI: 07–31%	20%, 95% CI: 13–29%	4%, 95% CI: 3–6%
	Overall	18%, 95% CI: 07–31%	14%, 95% CI: 10–20%	NA
Region	Sub-Sahara	31%, 95% CI: 23–40%	30%, 95% CI:22–40%	24%, 95% CI:11–40%
	North Africa	44%, 95% CI: 34–53%	55%, 95% CI: 46–65%	31%, 95% CI: 18–47%
Instruments	GAD-7/PHQ-9	34%, 95% CI: 25–43%	43%, 95% CI: 33–54%	NA
	DASS-22	44%, 95% CI: 31–57%	43%, 95% CI: 29–59%	

Note: CI = Confidence Interval.

**Table A3:** Studies included in this meta-analysis.

Authors & Year	Data Collection Time	Country	Population	Sample Size	Outcome	Instrument
Agberotimi et al., 2020	20 March–19 April	Nigeria	GHCW, GP	382, 502	ANX, DEP, INS	GAD7, PHQ9, ISI
Arafa et al., 2021	16 April–30 April	Egypt	GP	1629	ANX, DEP	DASS21
Aylie et al., 2020	15 May–15 June	Ethiopia	GP	322	ANX, DEP	DASS21
Cenat et al., 2020	15 March–15 May	Togo, RDC, Rwanda	GP	242, 626, 174	ANX	HSCL
Elhadi et al., 2020 a	20 April–1 May	Libya	Student	2430	ANX, DEP	GAD7, PHQ9
Elhadi et al., 2020 b	18 April–28 April	Libya	FHCW	154	ANX, DEP	HADS
Elhadi et al., 2020 c	18 April–28 April	Libya	GHCW	745	ANX, DEP	HADS
Elkholy et al., 2020	April–May	Egypt	FHCW	457	ANX, DEP, INS	GAD7, PHQ9, ISI
Essangri et al., 2021	8 April–18 April	Morocco	Student	549	ANX, DEP, INS	GAD7, PHQ9, ISI
Idrissi et al., 2020	1 April–1 May	Morocco	GP	846	ANX, DEP, INS	HARS, BDI, AIS
Kassaw 2020	10 March–30 March	Ethiopia	GP	382	ANX, DEP	DASS21
Keubo et al., 2020	5 April–19 April	Cameroon	FHCW	292	ANX, DEP	HAD
Kibret et al., 2020	15 May–15 June	Ethiopia	GHCW	305	ANX	GAD7
Kim et al., 2020	26 March–7 May	South Africa	GP	211	DEP	CESD10
Kounou et al., 2020	18 May	Togo	GHCW	62	ANX, DEP	GAD7, PHQ9
Mboua et al., 2020	5 April–19 April	Cameroon	GHCW	332	ANX, DEP	HADS
Mekonen et al., 2020	25 Sepetember–20 October	Ethiopia	GHCW	302	ANX, DEP	DASS21
Mulatu et al., 2020	1 August–30 August	Ethiopia	GHCW	420	ANX, DEP, INS	GAD7, PHQ9, ISI
Naidoo et al., 2020	6 March *	South Africa	GHCW	150	ANX, DEP	GAD7, PHQ9
Omar et al., 2021	30 March–30 June	Egypt	GP (M, F)	217, 479	ANX, DEP	GAD7, PHQ9
Rakhmanov et al., 2020	21 April *	Nigeria	GP	69	ANX	SRQ21
Sagaon-Teyssier et al., 2020	6 April–6 May	Mali	GHCW	135	ANX, DEP, INS	GAD7, PHQ9, ISI
Sediri et al., 2020	25 April–6 May	Tunisia	GP	751	ANX, DEP	DASS21
Shuwiekh et al., 2020	28 April–25 May	Egypt	GP	255	ANX, DEP	GAD7, PHQ9
Souilhi et al., 2020	22 September **	Tunisia	GHCW	48	ANX, DEP, INS	GAD7, PHQ9, ISI
Teshome et al., 2020	20 May–20 June	Ethiopia	GHCW	798	ANX	GAD7
Yitayih et al., 2020	22 March–28 March	Ethiopia	GHCW	249	INS	ISI
Youssef et al., 2020	1 April–30 April	Egypt	GP	540	ANX, DEP, INS	DASS21, ISI

Note: GHCW = general healthcare workers, FHCW = frontline healthcare workers, GP = general population, ANX = anxiety, DEP = depression, INS = insomnia. * Paper submission date, as no data collection time was reported; ** paper online date, as no data collection time was reported.
